# Supplementary material for: Phytochemical Profile of Trigonella caerulea (Blue Fenugreek) Herb and Quantification of Aroma-Determining Constituents
Source: Plants (Basel). 2023 Mar 3;12(5):1154. doi: 10.3390/plants12051154 (PMC10005085; doi:10.3390/plants12051154)
Supplement: Supplementary file 1 [file plants-12-01154-s001.zip › plants-2244498-supplementary.pdf]

Supporting Information

# Phytochemical Profile of *Trigonella caerulea* (Blue Fenugreek) Herb and Quantification of Aroma-Determining Constituents

Arpine Ayvazyan [1], Thomas Stegemann [1,2], Mayra Galarza Pérez[1], Manuel Pramsohler [3] and Serhat Sezai Çiçek [1,*]

[1] Department of Pharmaceutical Biology, Kiel University, Gutenbergstraße 76, 24118 Kiel, Germany

[2] Botanical Institute and Botanic Gardens, Kiel University, Am Botanischen Garten 1-9, 24118 Kiel, Germany

[3] Laimburg Research Centre, 39040 Auer/Ora, BZ, Italy

* Corresponding author. *E-mail address*: scicek@pharmazie.uni-kiel.de

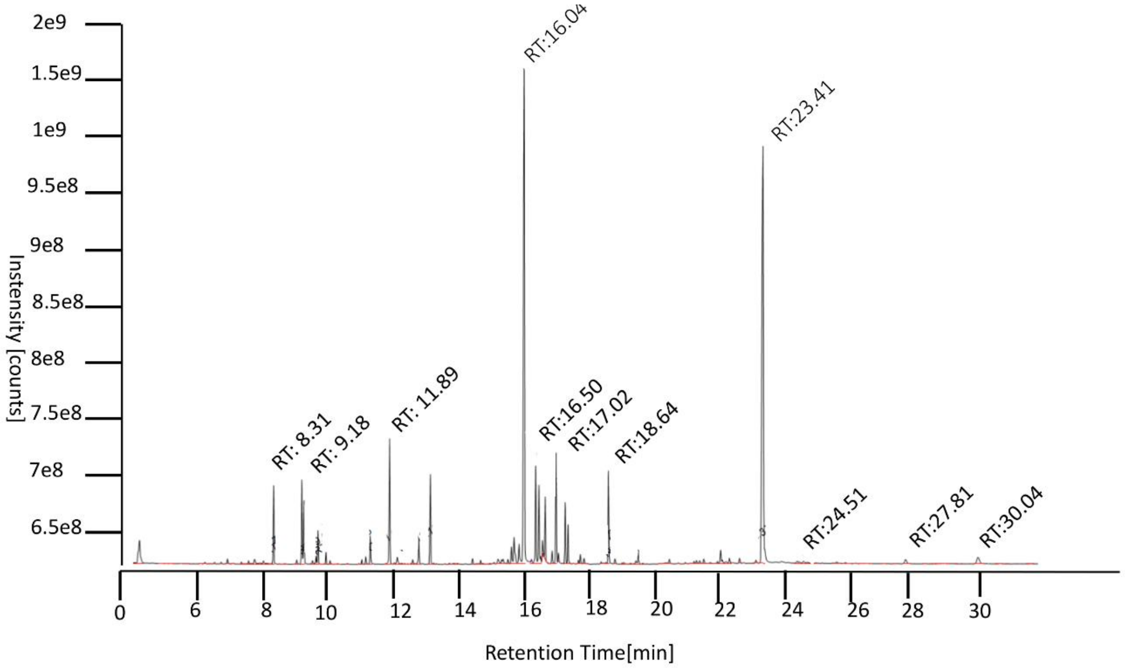


**Figure S1.** Total ion GC-MS chromatogram from untargeted profiling of primary metabolites. Column: ThermoFisher TG-5SilMS (30 m × 0.25 mm × 0.25 µm). GC conditions: 100°C hold for 5 min, 25°C/min to 160°C hold 1 min, 10°C/min to 300°C hold for 12 min. MS parameters: Fullscan 50-500 m/z. Ion source temperature: 280°C.

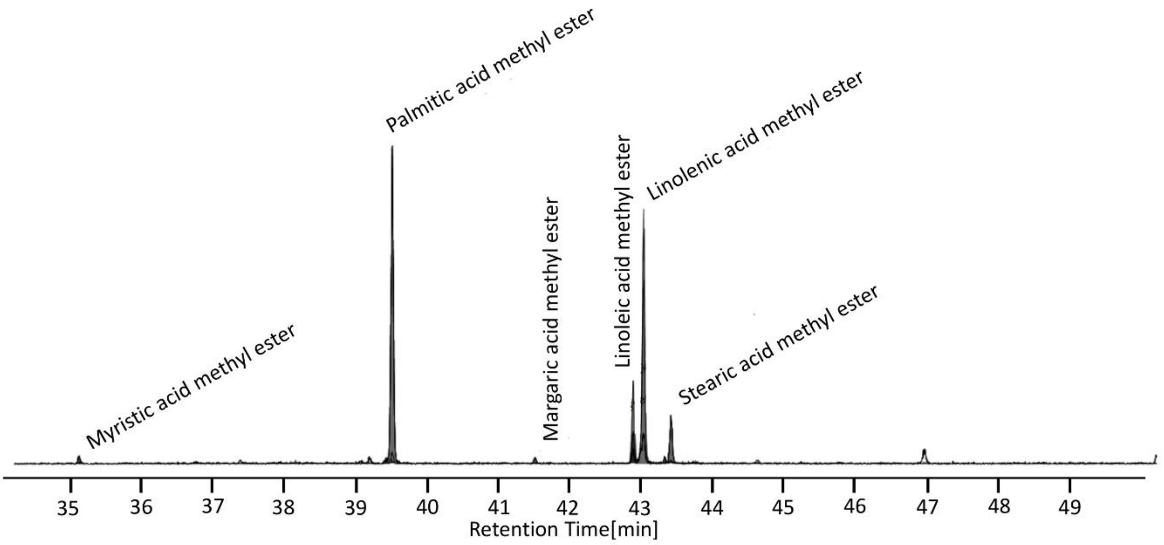


**Figure S2.** GC-MS chromatogram of fatty acid analysis. Column: ThermoFisher TG-5SilMS (30 m × 0.25 mm × 0.25 µm). GC conditions: 50°C for 5 min and heated with 5°C/min to 160°C hold 1 min, 5°C/min to 300°C and hold for 5 min. MS parameters: Full Scan from 50-500 m/z, ion source temperature: 280°C.

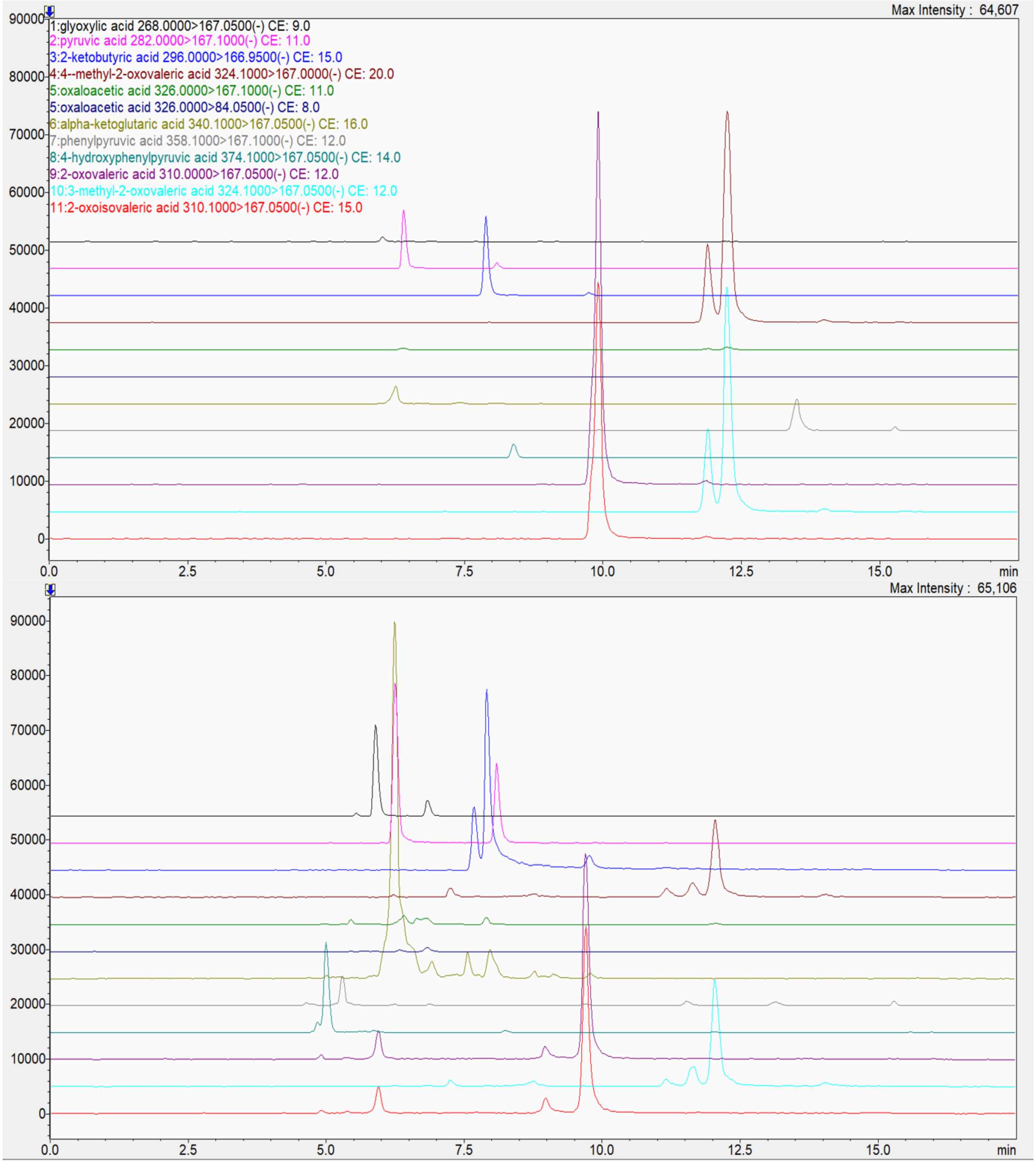


**Figure S3.** UHPLC-MS/MS chromatogram of standard solution (top) and sample (bottom). Column: Phenomenex Kinetex Biphenyl (100 × 2.1 mm, 1.7 µM). LC conditions: 50 mM formic acid in water (A) and acetonitrile (B). Gradient: 0 min (10% B), 2 min (10% B), 2.5 min (35% B), 13.5 min (40% B), 13.6 min (95% B), 17.5 min (95% B). Post run: 4 min. Column temperature: 40°C. Flow rate: 0.3 mL/min. Injection: 5 µL. MS conditions: Nebulizing gas flow: 3 L/min, DL temperature 250°C, heat block temperature: 400°C, drying gas flow: 15 L/min. Collision energies and transitions used for quantification are depicted in the figures.

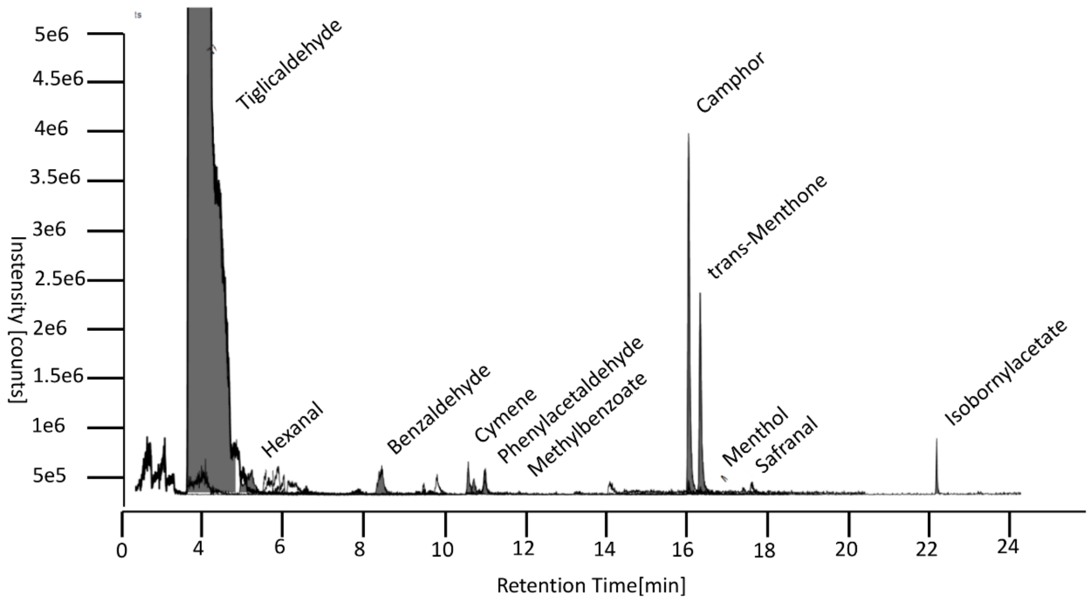


**Figure S4.** Total ion chromatogram of headspace GC-MS analysis. Column: ThermoFisher TG-5SilMS (30 m × 0.25 mm × 0.25 µm). GC conditions: 35°C hold 1 min, 5°C/min to 120°C and hold 1 min, 30°C/min to 300°C and hold 1 min. The MS parameters were as follows: 43-300 m/z scan with MS Source at 280°C.

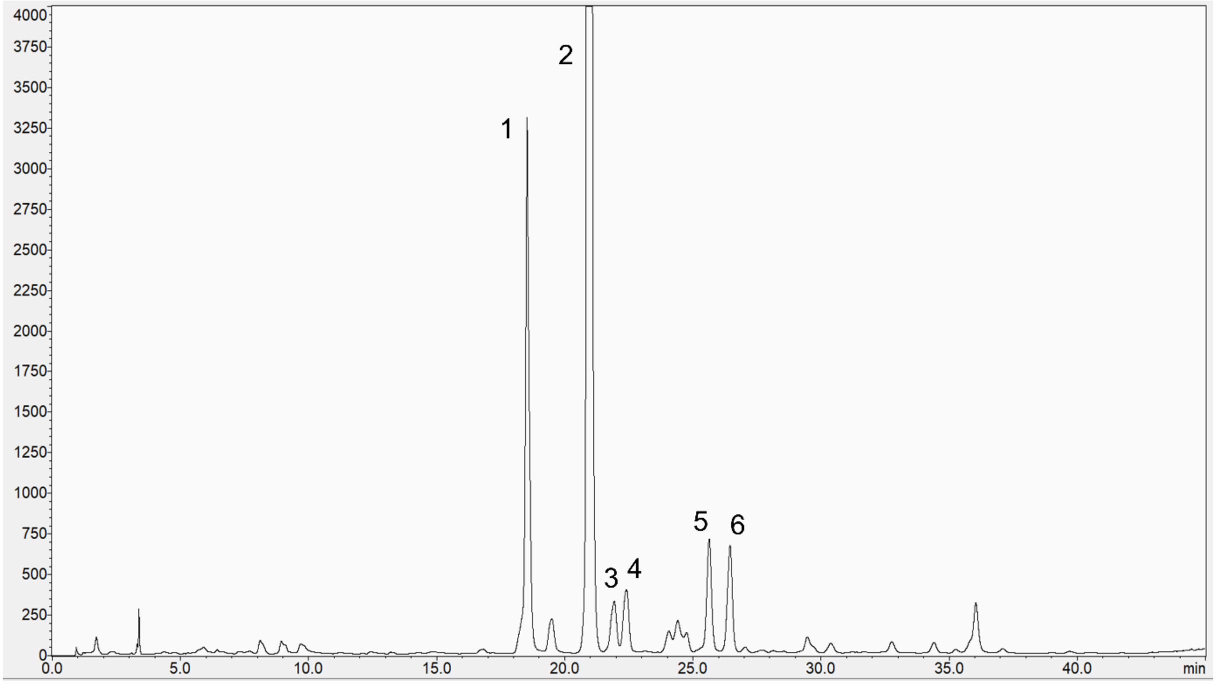


**Figure S5.** UHPLC-PDA chromatogram of blue fenugreek methanol extract at a wavelength of 350 nm. Column: Phenomenex Luna Omega Polar (100 × 2.1 mm, 1.6 µm particle size). Solvent system: 1% formic acid in water (A) and 1% formic acid in acetonitrile (B). Gradient: 0 min (5% B), 10 min (10% B), 40 min (20% B), 45 min (32% B), 60 min (95% B), 65 min (95% B). Post run: 10 min. Column temperature: 35°C. Flow rate: 0.3 mL/min. Injection: 2 µL. Compounds: Quercetin 3-O-(2″-O-α-L-rhamnopyranosyl)-β-D-glucopyranoside 7-O-β-D-rhamnopyranoside (**1**), kaempferol 3-O-(2″-O-α-L-rhamnopyranosyl)-β-D-glucopyranoside 7-O-β-D-rhamnopyranoside (**2**), quercetin 3-O-(2″-O-α-L-rhamnopyranosyl)-β-D glucopyranoside (**3**), quercetin 3-O-β-D-glucopyranoside 7-O-β-D-rhamnopyranoside (**4**), kaempferol 3-O-(2″-O-α-L-rhamnopyranosyl)-β-D glucopyranoside (**5**), and kaempferol 3-O-β-D glucopyranoside 7-O-β-D-rhamnopyranoside (**6**).

**Table S1**

List of compounds obtained from untargeted profiling of primary metabolites. All values were obtained from the total ion chromatogram. Relative peaks areas are calculated from identified components.

| Peak Name | $t_{Ret}$ min | TC1* rel. area (%) | TC2* rel. area (%) | TC3* rel. area (%) | SI | RSI |
|---|---|---|---|---|---|---|
| Lactic Acid 2 TMS | 6.48 | 0.06 | 0.06 | 0.06 | 835 | 920 |
| Glycolic Acid 2 TMS | 6.68 | 0.06 | 0.05 | 0.05 | 825 | 887 |
| Valine TMS | 6.88 | 0.18 | 0.20 | 0.22 | 835 | 871 |
| Alanine 2 TMS | 7.06 | 0.03 | 0.03 | 0.04 | 710 | 829 |
| Leucine TMS | 7.72 | 0.26 | 0.29 | 0.32 | 750 | 773 |
| Isoleucine TMS | 7.99 | 0.16 | 0.18 | 0.20 | 788 | 821 |
| Malonic Acid 2 TMS | 8.31 | 2.75 | 3.05 | 2.46 | 901 | 901 |
| Serine 2 TMS | 9.02 | 0.17 | 0.19 | 0.21 | 879 | 900 |
| Glycerol 3 TMS | 9.18 | 3.13 | 3.47 | 3.86 | 929 | 931 |
| Phosphate 3 TMS | 9.25 | 2.33 | 2.59 | 2.87 | 938 | 941 |
| Threonine 2 TMS | 9.51 | 0.16 | 0.18 | 0.20 | 824 | 876 |
| Succinic Acid 2 TMS | 9.68 | 1.16 | 1.29 | 1.43 | 930 | 935 |
| Glyceric Acid 3 TMS | 9.92 | 0.42 | 0.47 | 0.52 | 890 | 899 |
| Fumaric Acid 2 TMS | 10.05 | 0.12 | 0.13 | 0.15 | 902 | 936 |
| Caproic acid 3-hydroxy, 2TMS | 11.03 | 0.17 | 0.19 | 0.21 | 710 | 756 |
| Malic acid 3TMS | 11.89 | 4.86 | 5.39 | 4.77 | 923 | 928 |
| Threitol 4 TMS | 12.13 | 0.42 | 0.47 | 0.52 | 707 | 821 |
| 4-Hydroxybenzoic acid 2 TMS | 13.59 | 0.02 | 0.02 | 0.02 | 754 | 795 |
| Pinitol 5 TMS | 16.04 | 28.25 | 31.36 | 30.66 | 943 | 944 |
| Fructose MOX, 5 TMS Isomer 1 | 16.40 | 4.16 | 2.81 | 2.77 | 925 | 926 |
| Fructose MOX, 5 TMS Isomer 2 | 16.50 | 3.41 | 3.79 | 3.34 | 932 | 932 |
| Glucose MOX 5 TMS | 16.69 | 2.38 | 2.64 | 2.93 | 919 | 919 |
| Mannitol 6 TMS | 17.02 | 4.62 | 5.13 | 3.87 | 946 | 946 |
| Gluconic Acid 6 TMS | 17.71 | 0.16 | 0.18 | 0.20 | 828 | 903 |
| Palmitic Acid TMS | 17.88 | 0.23 | 0.26 | 0.28 | 864 | 884 |
| Ferulic acid 2 TMS | 18.48 | 0.05 | 0.06 | 0.06 | 798 | 872 |
| Myo-Inositol 6 TMS | 18.64 | 3.91 | 4.34 | 3.33 | 946 | 964 |
| Galactose MOX, 6 TMS | 18.84 | 0.22 | 0.24 | 0.27 | 820 | 868 |
| Linoleic acid TMS | 19.48 | 0.18 | 0.20 | 0.22 | 842 | 930 |
| Linolenic acid TMS | 19.56 | 0.47 | 0.52 | 0.58 | 884 | 888 |
| Stearic acid TMS | 19.70 | 0.05 | 0.06 | 0.06 | 716 | 802 |
| Methylhydroxycinnamate TMS | 22.37 | 0.25 | 0.28 | 0.31 | 667 | 767 |
| Saccharose 8 TMS | 23.41 | 33.68 | 28.41 | 31.55 | 946 | 961 |
| Aucubin 6 TMS | 24.54 | 0.11 | 0.12 | 0.14 | 780 | 826 |
| Galacitinol 9 TMS | 27.81 | 0.47 | 0.52 | 0.58 | 861 | 884 |
| Glycerol-Galactopyranoside 6 TMS | 30.04 | 0.94 | 1.04 | 0.81 | 832 | 877 |

* TC1 means *T. caerulea* sample 1, TC2 means *T. caerulea* sample 2, TC3 means *T. caerulea* sample 3.

**Table S2**

NMR data for compound **1** in DMSO-$d_6$ (400 MHz, $\delta$ in ppm, $J$ in Hz).

| Position | $^1$H NMR | $^{13}$C NMR | HMBC | H,H COSY |
|---|---|---|---|---|
| aglycone | | | | |
| 2 | | 156.7 | | |
| 3 | | 133.2 | | |
| 4 | | 177.5 | | |
| 5 | | 160.9 | | |
| 6 | 6.42 (s) | 99.4 | C-5, C-7, C-8, C-10 | |
| 7 | | 161.6 | | |
| 8 | 6.79 (d, 2.0) | 94.2 | C-6, C-7, C-9, C-10, | |
| 9 | | 155.9 | | |
| 10 | | 105.6 | | |
| 1´ | | 121.0 | | |
| 2´ | 7.58 (d, 2.2) | 116.1 | C-2, C-3´, C-4´, C-6´ | |
| 3´ | | 145.0 | | |
| 4´ | | 148.7 | | |
| 5´ | 6.85 (d, 8.5) | 115.2 | C-3´, C-4´, C-6´ | |
| 6´ | 7.64 (dd, 8.4, 2.2) | 121.8 | C-2, C-2´, C-4´ | |
| OH-5 | 12.64 | | | |
| OH-3´ | 9.23 | | | |
| OH-4´ | 9.88 | | | |
| 3-O-glucose | | | | |
| 1´´ | 5.65 (d, 7.7) | 98.3 | C-3 | H-2´´ |
| 2´´ | 3.51(m) | 77.4 | C-1´´, C-3´´, C-1´´´´ | H-1´´ |
| 3´´ | 3.42 (m) | 77.3 | | |
| 4´´ | 3.09 (m) | 70.3 | | |
| 5´´ | 3.08 (m) | 77.6 | | |
| 6´´ | 3.29 (m)<br>3.57 (dd, 12.3, 5.7) | 60.9 | | |
| 7-O-rhamnose | | | | |
| 1´´´ | 5.55 (brs) | 98.4 | C-7, C-3´´´ | H-2´´´ |
| 2´´´ | 3.84 (m) | 69.8 | | H-3´´´ |
| 3´´´ | 3.63 (m) | 70.2 | | H-2´´´, H-4´´´ |
| 4´´´ | 3.32 (m) | 71.6 | | H-5´´´ |
| 5´´´ | 3.44 (m) | 70.1 | | H-6´´´ |
| 6´´´ | 1.11 (d, 6.1) | 18.0 | C-4´´´, C-5´´´ | H-5´´´ |
| 2´´-O-rhamnose | | | | |
| 1´´´´ | 5.07 (brs) | 100.5 | C-2´´, C-3´´´´, C-5´´´´ | H-2´´´´ |
| 2´´´´ | 3.73 (m) | 70.6 | | H-3´´´´ |
| 3´´´´ | 3.46 (m) | 70.7 | C-4´´´´ | H-2´´´´, H-4´´´´ |
| 4´´´´ | 3.12 (m) | 71.9 | | H-5´´´´ |
| 5´´´´ | 3.71 (m) | 68.3 | | H-3´´´´ |
| 6´´´´ | 0.77 (d, 6.3) | 17.3 | C-4´´´´, C-5´´´´ | H-5´´´´ |

**Table S3**

NMR data for compound **2** in DMSO-$d_6$ (400 MHz, $\delta$ in ppm, $J$ in Hz).

| Position | $^1$H NMR | $^{13}$C NMR | HMBC | H,H COSY |
|---|---|---|---|---|
| aglycone | | | | |
| 2 | | 156.7 | | |
| 3 | | 133.0 | | |
| 4 | | 177.6 | | |
| 5 | | 160.9 | | |
| 6 | 6.44 (d, 2.2) | 99.5 | C-5, C-7, C-8, C-10, | H-8 |
| 7 | | 161.6 | | |
| 8 | 6.83 (d, 2.0) | 94.5 | C-6, C-7, C-9, C-10 | H-6 |
| 9 | | 156.0 | | |
| 10 | | 105.7 | | |
| 1´ | | 120.8 | | |
| 2´ | 8.08 (d, 9.0) | 130.9 | C-2, C-4´ | H-3´ |
| 3´ | 6.89 (d, 9.0) | 115.2 | C-1´, C-4´ | H-2´ |
| 4´ | | 160.1 | | |
| 5´ | 6.89 (d, 9.0) | 115.2 | C-1´, C-4´ | H-6´ |
| 6´ | 8.08 (d, 9.0) | 130.9 | C-2, C-4´ | H-5´ |
| OH-5 | 12.62 | | | |
| OH-4´ | 10.26 | | | |
| 3-O-glucose | | | | |
| 1´´ | 5.66 (d, 7.0) | 98.3 | C-3 | H-2´´ |
| 2´´ | 3.44 (m) | 77.4 | C-3´´ | H-1´´ |
| 3´´ | 3.39 (m) | 77.3 | | |
| 4´´ | 3.09 (m) | 70.3 | | |
| 5´´ | 3.08 (m) | 77.6 | | |
| 6´´ | 3.28 (m)<br>3.55 (dd, 11.3, 5.6) | 60.8 | | |
| 7-O-rhamnose | | | | |
| 1´´´ | 5.55 (d, 1.5) | 98.4 | C-7, C-2´´´ | |
| 2´´´ | 3.84 (m) | 69.8 | | |
| 3´´´ | 3.63 (m) | 70.2 | | |
| 4´´´ | 3.31 (m) | 71.6 | | |
| 5´´´ | 3.42 (m) | 70.1 | C-4´´´, C-6´´´ | |
| 6´´´ | 1.11 (d, 6.2) | 18.0 | C-4´´´, C-5´´´ | |
| 2´´-O-rhamnose | | | | |
| 1´´´´ | 5.07 (d, 0.9) | 100.7 | C-2´´, C-3´´´´, C-5´´´´ | H-2´´´´ |
| 2´´´´ | 3.73 (m) | 70.6 | | H-3´´´´ |
| 3´´´´ | 3.46 (m) | 70.5 | | H-4´´´´ |
| 4´´´´ | 3.13 (m) | 71.8 | C-6´´´´ | H-3´´´´, H-5´´´´ |
| 5´´´´ | 3.70 (m) | 68.4 | C-4´´´´ | H-4´´´´ |
| 6´´´´ | 0.75 (d, 6.2) | 17.3 | C-4´´´´, C-5´´´´ | H-5´´´´ |

**Table S4**
NMR data for compound **3** in DMSO-$d_6$ (400 MHz, $\delta$ in ppm, $J$ in Hz).

| Position | $^{1}$H NMR | $^{13}$C NMR | HMBC | H,H COSY |
|---|---|---|---|---|
| aglycone | | | | |
| 2 | | 157.0 | | |
| 3 | | 133.1 | | |
| 4 | | 177.5 | | |
| 5 | | 161.6 | | |
| 6 | 6.12 (s) | 99.6 | | H-8 |
| 7 | | 166.5 | | |
| 8 | 6.33 (d, 8.1) | 94.2 | | H-6 |
| 9 | | 156.9 | | |
| 10 | | 103.6 | | |
| 1´ | | 121.3 | | |
| 2´ | 7.52 (d, 2.2) | 116.2 | C-3´ | |
| 3´ | | 145.5 | | |
| 4´ | | 156.1 | | |
| 5´ | 6.87 (d, 8.6) | 115.6 | C-1´, C-3´ | H-6´ |
| 6´ | 7.60 (dd, 10.4, 6.2) | 122.1 | C-4´ | H-5´ |
| OH-5 | 12.66 | | | |
| OH-3´ | 8.46 | | | |
| OH-4´ | 8.24 | | | |
| 3-O-glucose | | | | |
| 1´´ | 5.66 (d, 7.4) | 98.8 | C-3 | H-2´´, H-3´´ |
| 2´´ | 3.50(m) | 78.0 | C-1´´´ | H-3´´ |
| 3´´ | 3.39 (m) | 77.8 | C-4´´ | H-4´´ |
| 4´´ | 3.09 (m) | 70.7 | | H-3´´ |
| 5´´ | 3.08 (m) | 77.7 | | H-6´´ |
| | 3.30 (m) | | C-2´´ | |
| 6´´ | 3.56 (dd, 12.3, 5.8) | 61.4 | | |
| 2´´-O-rhamnose | | | | |
| 1´´´ | 5.08 (brs) | 100.9 | C-2´´, C-3´´´ | H-5´´´ |
| 2´´´ | 3.74 (m) | 70.8 | | H-3´´´, H-4´´´ |
| 3´´´ | 3.46 (m) | 71.0 | C-4´´´ | H-4´´´, H-5´´´ |
| 4´´´ | 3.13 (m) | 72.3 | C-2´´´, C-5´´´ | H-3´´´, H-5´´´ |
| 5´´´ | 3.72 (m) | 68.7 | C-2´´´, C-4´´´ | H-4´´´, H-6´´´ |
| 6´´´ | 0.78 (d, 6.1) | 17.7 | C-4´´´, C-5´´´ | H-2´´´ |

**Table S5**

NMR data for compound **4** in DMSO-$d_6$ (400 MHz, $\delta$ in ppm, $J$ in Hz).

| Position | $^{1}$H NMR | $^{13}$C NMR | HMBC | H,H COSY |
|---|---|---|---|---|
| aglycone | | | | |
| 2 | | 157.1 | | |
| 3 | | 134.0 | | |
| 4 | | 172.4 | | |
| 5 | | 161.3 | | |
| 6 | 6.44 (s) | 99.8 | C-5, C-7, C-10 | |
| 7 | | 162.0 | | |
| 8 | 6.80 (d, 2.0) | 94.8 | C-7 | H-6 |
| 9 | | 155.9 | | |
| 10 | | 106.0 | | |
| 1´ | | 121.2 | | |
| 2´ | 7.63 (d, 2.2) | 116.7 | C-2, C-1´, C-3´, C-6´ | |
| 3´ | | 145.4 | | |
| 4´ | | 149.4 | | |
| 5´ | 6.85 (d, 9.0) | 115.7 | C-1´, C-3´, C-4´ | H-6´ |
| 6´ | 7.61 (d, 2.3) | 122.2 | C-2, C-4´ | |
| OH-5 | 12.62 | | | |
| OH-3´ | 8.41 | | | |
| OH-4´ | 8.29 | | | |
| 7-O-rhamnose | | | | |
| 1´´ | 5.56 (brs) | 98.8 | C-7, C-3´´ | H-2´´ |
| 2´´ | 3.84 (m) | 70.3 | | |
| 3´´ | 3.63 (m) | 70.7 | C-4´´ | |
| 4´´ | 3.29 (m) | 71.9 | C-3´´ | |
| 5´´ | 3.43 (m) | 70.5 | C-4´´ | |
| 6´´ | 1.12 (d, 6.3) | 18.4 | C-4´´, C-5´´ | H-5´´ |
| 3-O-glucose | | | | |
| 1´´´ | 5.50 (d, 7.7) | 101.5 | C-3 | H-2´´´, H-3´´´ |
| 2´´´ | 3.24 (m) | 74.5 | C-3´´´ | |
| 3´´´ | 3.26 (m) | 77.0 | | H-1´´´ |
| 4´´´ | 3.09 (m) | 70.4 | C-5´´´ | |
| 5´´´ | 3.08 (m) | 78.1 | | |
| 6´´´ | 3.32 (m)<br>3.65 (d, 3.2) | 61.4 | | |

**Table S6**
NMR data for compound **5** in DMSO-$d_6$ (400 MHz, $\delta$ in ppm, $J$ in Hz).

| Position | $^1$H NMR | $^{13}$C NMR | HMBC | H,H COSY |
|---|---|---|---|---|
| aglycone | | | | |
| 2 | | 156.6 | | |
| 3 | | 132.5 | | |
| 4 | | 177.0 | | |
| 5 | | 161.1 | | |
| 6 | 6.09 (d, 2.2) | 99.4 | C-5, C-7, C-8, C-10 | |
| 7 | | 166.0 | | |
| 8 | 6.32 (d, 2.0) | 94.0 | C-6, C-7, C-9, C-10 | |
| 9 | | 160.1 | | |
| 10 | | 103.0 | | |
| 1´ | | 120.9 | | |
| 2´ | 8.02 (d, 9.0) | 130.6 | C-1, C-9 | |
| 3´ | 6.88 (d, 2.0) | 115.1 | C-9 | |
| 4´ | | 155.5 | | |
| 5´ | 6.88 (d, 2.0) | 115.1 | C-9 | |
| 6´ | 8.02 (d, 9.0) | 130.6 | C-1, C-9 | |
| OH-5 | 12.62 | | | |
| OH-4´ | 8.47 | | | |
| 3-O-glucose | | | | |
| 1´´ | 5.66 (d, 7.1) | 98.3 | C-3, C-2´´ | H-2´´ |
| 2´´ | 3.44 (m) | 77.5 | | H-1´´, H-6´´ |
| 3´´ | 3.38 (m) | 77.4 | | H-2´´, H-5´´ |
| 4´´ | 3.09 (m) | 70.2 | | |
| 5´´ | 3.08 (m) | 77.3 | | H-3´´ |
| 6´´ | 3.30 (m)<br>3.56 (dd, 11.8, 6.8) | 61.2 | | |
| 2´´-O-rhamnose | | | | |
| 1´´´ | 5.08 (s) | 100.6 | C-2´´, C-2´´´, C-5´´´ | H-2´´´ |
| 2´´´ | 3.74 (m) | 70.5 | | H-4´´´ |
| 3´´´ | 3.46 (m) | 70.6 | | H-4´´´, H-5´´´ |
| 4´´´ | 3.12 (m) | 71.8 | | H-3´´´, H-5´´´ |
| 5´´´ | 3.72 (m) | 68.8 | C-2´´´, C-4´´´ | H-1´´´, H-3´´´, H-4´´´ |
| 6´´´ | 0.76 (d, 6.1) | 17.7 | C-4´´´, C-5´´´ | H-5´´´ |

**Table S7**
NMR data for compound **6** in DMSO-$d_6$ (400 MHz, $\delta$ in ppm, $J$ in Hz).

| Position | $^{1}$H NMR | $^{13}$C NMR | HMBC | H,H COSY |
|---|---|---|---|---|
| aglycone | | | | |
| 2 | | 157.2 | | |
| 3 | | 133.8 | | |
| 4 | | 178.0 | | |
| 5 | | 161.4 | | |
| 6 | 6.45 (d, 2.2) | 99.8 | C-5, C-7, C-8, C-10 | H-8 |
| 7 | | 162.0 | | |
| 8 | 6.84 (d, 2.0) | 94.9 | C-6, C-7, C-9, C-10 | H-6 |
| 9 | | 156.4 | | |
| 10 | | 106.1 | | |
| 1´ | | 120.9 | | |
| 2´ | 8.09 (d, 9.0) | 131.5 | C-2, C-3´, C-4´ | H-3´ |
| 3´ | 6.89 (d, 9.0) | 115.7 | C-1´, C-4´ | H-2´ |
| 4´ | | 160.9 | | |
| 5´ | 6.89 (d, 9.0) | 115.7 | C-2, C-4´, C-6´ | H-6´ |
| 6´ | 8.09 (d, 9.0) | 131.5 | C-1´, C-4´ | H-5´ |
| OH-5 | 12.62 | | | |
| OH-4´ | 8.47 | | | |
| 7-O-rhamnose | | | | |
| 1´´ | 5.56 (d, 1.2) | 98.8 | C-7, C-5´´ | |
| 2´´ | 3.85 (m) | 70.3 | | |
| 3´´ | 3.63 (m) | 70.7 | | |
| 4´´ | 3.32 (m) | 72.0 | C-5´´ | |
| 5´´ | 3.43 (m) | 70.5 | | |
| 6´´ | 1.12 (d, 6.2) | 18.4 | C-4´´, C-5´´ | H-5´´ |
| 3-O-glucose | | | | |
| 1´´´ | 5.49 (d, 7.3) | 101.2 | C-3 | H-2´´´, H-3´´´ |
| 2´´´ | 3.20 (m) | 74.6 | | |
| 3´´´ | 3.22 (m) | 76.9 | | |
| 4´´´ | 3.10 (m) | 70.4 | | |
| 5´´´ | 3.09 (m) | 78.0 | | |
| 6´´´ | 3.33 (m)<br>3.57 (d, 11.4) | 61.3 | | |
